# FOOD TALK AS AGING TALK: LISTENING TO OLDER ITALIANS

**DOI:** 10.1093/geroni/igad104.2715

**Published:** 2023-12-21

**Authors:** Anne Barrett, Monica Consolandi, Eliana Fattorini

**Affiliations:** Florida State University, Tallahassee, Florida, United States; Bruno Kessler Foundation, Trento, Trentino-Alto Adige, Italy; University of Trento, Trento, Trentino-Alto Adige, Italy

## Abstract

An extensive literature in gerontology examines older adults’ diets and their effects on health and longevity. Much less research examines how food, including experiences of its scarcity and plentitude across the life course, shape older adults’ constructions of their own aging. Our study addressed this topic using semi-structured interviews conducted in a mid-sized northern Italian city in 2018-2019 (n=28) and 2022 (n=23). Analyses revealed that participants used vivid references to food – a topic that was not the focus of any interview questions – to illustrate their experiences of aging. Participants’ food talk referenced different tenses of time, including durable food memories from the distant past and more ephemeral food activities and concerns of the present. Participants also referred to food when making different comparisons that shaped assessments of their own aging experiences, including comparisons with older family members in the past and age peers in the present. Food figured into participants’ discussions of aging in four ways: food as anchors for childhood memories, intergenerational comparisons of food’s plentitude versus scarcity in later life, food choices to support aging well, and food sharing to sustain social relationships. Our study illustrates the use of food a lens for understanding individuals’ aging experiences, as embedded in historical times that give them meaning.

